# Response mechanisms of 3 typical plants nitrogen and phosphorus nutrient cycling to nitrogen deposition in temperate meadow grasslands

**DOI:** 10.3389/fpls.2023.1140080

**Published:** 2023-07-07

**Authors:** Yang Zhang, Qing Zhang, Wenjun Yang, Yan Zhang, Ning Wang, Peixian Fan, Chao You, Linqian Yu, Qun Gao, Hui Wang, Peiming Zheng, Renqing Wang

**Affiliations:** ^1^ Institute of Ecology and Biodiversity, School of Life Sciences, Shandong University, Qingdao, China; ^2^ Shandong Provincial Engineering and Technology Research Center for Vegetation Ecology, Shandong University, Qingdao, China; ^3^ Qingdao Forest Ecology Research Station of National Forestry and Grassland Administration, Shandong University, Qingdao, China

**Keywords:** litter decomposition, nitrogen deposition, nutrient resorption, temperate meadow steppe, nutrient uptake

## Abstract

The increase of nitrogen (N) deposition and the diversity of its components lead to significant changes in the structure and function of temperate meadow steppe, which could affect plant nutrient uptake, nutrient resorption and litter decomposition, thus affecting the biogeochemical cycle process. The distribution and metabolism of nitrogen and phosphorus in plants determine the growth process and productivity of plants. Plant nutrient uptake, nutrient resorption and litter decomposition play an important role in the nutrient cycling process of ecosystem. This study closely combined these three processes to carry out experiments with different nitrogen dosages and types, and systematically explored the response of nitrogen and phosphorus nutrient cycling to nitrogen deposition. The results showed that nitrogen deposition can greatly affect ecosystem nutrient cycle of nitrogen and phosphorus. Firstly, Nitrogen deposition has significant effect on plant nutrient uptake. Nitrogen uptake of stems and leaves increased with the increase of nitrogen addition dosage, while phosphorus uptake of stems and leaves showed a downward trend or no significant effect. Besides, nitrogen addition type had a significant effect on nitrogen and phosphorus content of stems. Secondly, Nitrogen addition dosage had a significant effect on plant nutrient resorption, while nitrogen addition type had no significant effect on it. Thirdly, nitrogen deposition has significant effect on litter decomposition. With the increase of nitrogen addition dosage, the initial nitrogen content of litters increased and the decomposition rate of litters accelerated. Nitrogen application type had significant effect on stem litter decomposition. These results indicated that nitrogen deposition significantly affects plant nutrient cycling, and thus affects the structure and function of grassland ecosystem.

## 1 Introduction

There has been a dramatic increase in nitrogen (N) deposition in the global atmosphere due to increased human activity, in particular fossil fuel combustion and intensive farming (Galloway et al., 2008). N deposition has gradually become one of the driving factors of global change. N is one of the primary limiting nutrients in terrestrial ecosystems, and it is also one of the most important factors required for plant growth. At present, the increase of the nitrogen deposition in atmosphere has aroused the concern of scientists, and it has been proved that the increase of nitrogen deposition has caused many ecological problems (Sun et al., 2022). Atmospheric reactive nitrogen is deposited through both dry and wet pathways, including reduced NH_x_ and oxidized NO_y_ (Yu et al., 2019). In addition, nitrogen deposition was accompanied by ${\text{SO}}_{4}^{2-}$ deposition. The response of nutrient cycle to nitrogen deposition was also influenced by different nitrogen compound types (Dong et al., 2020).

Plant nutrient uptake refers to the process of transporting mineral nutrients absorbed by plant roots to the aboveground parts through xylem. The rate of nutrient uptake by the plant to the external environment is related to genetics, environmental nutrient concentration and plant size. The nutrient content of plants (plant element stoichiometry) represents nutrient uptake, and the measurements of nitrogen and phosphorus concentrations in organs are used to compare differences between plant species and differences within species (Guiz et al., 2018).

Nutrient resorption is a physiological process of transferring nutrients from senescent tissues to other living tissues (Aerts, 1996), which increases the residence time of nutrients in the plant, enabling rapid recycling of nutrients in the plant and the environment. Nutrient resorption efficiency (NuRE) and nutrient resorption proficiency (NuRP) are widely used measures for quantifying nutrient resorption (Zhang et al., 2020). NuRE is quantified by the percentage of nutrient resorbed in plant tissues, which is usually calculated from the nutrient concentration in the plant tissue during senescence (Killingbeck, 1984), which is highly dependent on leaf nutrient status globally and varies significantly between species and sites (Zhang et al., 2015). Nutrient proficiency refers to the degree to which the N concentration of a leaf decreases during aging (i.e., the lower the N concentration of a senescing leaf, the less N the leaf loses through defoliation and the higher the N resorption proficiency) (Oikawa et al., 2020). Global variations, including nitrogen deposition and precipitation changes, may modulate the nutrient resorption and distribution of plant (Van Heerwaarden et al., 2003). Previous studies have reported that the increase in nitrogen deposition significantly reduces the efficiency of nitrogen resorption (Gao et al., 2022). However, some studies have also shown that the addition of nitrogen did not change the nitrogen resorption efficiency (Zhao et al., 2020) or be enhanced (Birk and Vitousek, 1986; Yuan et al., 2005) with increasing supply of fertilizer in different habitats (Yuan et al., 2005). On the contrary, the effects of nitrogen enrichment on phosphorus (P) resorption varied greatly. There were positive, negative, and neutral effects of N enrichment on P resorption even in one ecosystem. (Su et al., 2021). In addition, revealing the response, mode and driving factors of plant nutrient status and chemical measurement to N enrichment is important for understanding how plants can adapt to human interference and the structure and function of the land ecosystem (Su et al., 2021). Therefore, the effects of adding N to the nutrient resorption of N and P remains to be further explored. Most previous studies focused on a single form of nitrogen addition (Lu et al., 2018), few studied the effects of nitrogen settlement from different types of nitrogen adding types on nutrient cycle.

The extent of nutrient uptake and resorption depends on the availability of nutrients in the environment and the costs involved in these processes(Wright and Westoby, 2003). In poor soils, plants have the characteristic of preferentially choosing resorption over active uptake of resources, whereas in nutrient-rich soils, the reverse is true. Specifically, because with the increase of soil nutrient availability, the capacity of nutrient uptake is lower than that of resorption, so the uptake strategy will be more popular than resorption (Kou et al., 2017). In contrast, species from nutrient-poor habitats are more likely to reduce nutrient loss by reclaiming most nutrients prior to shedding from aging organs (Killingbeck, 1996). In the context of consistent environmental nutrient availability, the costs of different plant nutrient acquisition strategies have not been effectively evaluated.

As a part of nutrient cycle, litter decomposition plays an important role in nutrient cycle of ecosystem. Aboveground litter is an important process of nutrient transfer from vegetation to soil and a key factor controlling nutrient cycling in terrestrial ecosystems. Research in the Netherlands showed that only 3 years of litter collection could decrease the available N in the soil by 8%, and the NH3 and N and NO concentrations were reduced by 56% and 31%, respectively (Li et al., 2015). Nutrient resorption affects the chemical composition of senescent plants and changes the litter quality and litter decomposition, finally affecting nutrient cycling. Previous studies have claimed that as nitrogen application increases, community litter nitrogen increases, thereby increasing the amount of nitrogen returned to the soil through litter production (Lü et al., 2013) and the early stage of litter decomposition is the stoichiometry between balancing microorganisms and litter (García-Palacios et al., 2016). In addition, litter decomposition is mainly controlled by the concentration of N in the litter, and the litter with high N content is more likely to decompose, so application of N may result in faster return of N to the soil (Zhang et al., 2015). However, some studies have shown that experimental nitrogen application accelerated and then slowed litter decomposition (Gill et al., 2021), and simulated N deposition significantly inhibited litter decomposition (Zhou et al., 2017). Among the factors influencing the decomposition rate, N and P concentration in the litter and the composition of structural carbohydrates are relatively important. Faster decomposition rates are generally associated with higher initial nitrogen concentrations and fewer cellulosic matter (Li et al., 2017). Litter quality (N content and C/N ratio), soil abiotic factors, and the composition of decomposer communities all affect decomposition rates (Hou et al., 2021). Nitrogen addition results in increased concentrations of N and P in plant leaves, thereby improving the quality of litter entering the soil (Li et al., 2022). The positive and negative effects of nitrogen deposition on the decomposition of litters have been reported, but there are few comparative studies on the decomposition quality of litters under the condition of nitrogen deposition by controlling environmental factors.

Different plant organs perform different functions, and the functional differences among organs lead to significant differences in stoichiometric characteristics (Liu et al., 2020; Liu et al., 2021). The study of nutrient resorptions in various plant organs is very important for understanding the nutritional economics of individual plants or communities (Brant and Chen, 2015). The distribution of nutrients to various organs depends on the physical structure and the biological function of the organs (Shi et al., 2020). Leaves have the highest resorption efficiency for most plants, followed by culms, due to the large number of structural compounds in culms that are not easily soluble (Aerts, 1996). Previous studies on nutrient resorption usually focused on leaves, but stems of grassland plants is also an important part of plants. Besides, in many terrestrial ecosystems, non-leaf organs usually make up a big percentage of total plant biomass, and they play an important role in plant nutrition economy (Li et al., 2015). Therefore, analyzing nutrient recycling patterns of different plant stems and leaves is helpful for us to further understand the optimization and improvement of the theoretical system of nutrient cycling process and mechanism in the ecosystem.

Temperate grassland constitutes the main body of grassland in China, which not only has an important economic and social benefit, but also is an important ecological barrier in northern China (Jin et al., 2014). Therefore, it is crucial to study the effect of nitrogen deposition on grassland ecosystem. The background value of N deposition in temperate meadow steppe ecosystem in Inner Mongolia was low, which was conducive to reflect the initial response of the ecosystem to N addition. Otherwise, previous studies on forest ecosystems have shown that N resorption efficiency (NRE) was higher in non-nitrogen-fixing species than in nitrogen-fixing species, phosphorus resorption efficiency (PRE) was lower in non-nitrogen-fixing species than in nitrogen-fixing species, indicating a significant effect of nitrogen-fixing capacity on N and P uptake (Jiang et al., 2019). Moreover, it was found that the availability of N could influence symbiotic N fixation by varying the biomass of grass legumes, and the contribution of N from symbiotic N fixation to the total N content of legumes (Vázquez et al., 2022). So, it is essential to explore the effects of N input on nutrient resorption in legumes for maintaining forage productivity and nutrient cycling in grasslands. Therefore, three common species in the temperate meadow steppe of Erguna, perennial poaceous plant *Leymus chinensis*, perennial legume *Thermopsis lanceolata*, and perennial rosaceae *Potentilla bifurc*a, were selected as our model plant species.

Specifically, we aim to address the following three questions: (1) How do nutrient uptake of plant stems and leaves respond to different nitrogen dosages and types? (2) Will the different N deposition dosages and types change the nutrient resorption of plant? (3) What’s the response mechanisms of litter decomposition to different N deposition in temperate meadow steppe ecosystems?

## 2 Materials and methods

### 2.1 Study site and plant material

Our research was carried out in the Erguna Forest-Steppe Ecoregion Research Station, which is located in the forest (N50°10′46.1′′, E119°22′56.4′′), which is located in Hulunbeier City, Inner Mongolia Autonomous Region. There is black calcareous soil which the pH is 7.0-8.0. Moreover, the contents of total nitrogen, available phosphorus, and organic matter in the topsoil (0-10 cm) were 1.8, 0.45, and 18.9 mg g^-1^ respectively. In addition, the region has a cold-temperate continental climate with an average annual precipitation of 336.5 mm (2000-2020) and annual temperature of –1.59°C. The low background values for nitrogen deposition in this region are favorable for nitrogen deposition simulation experiments.

### 2.2 Sample plot set-up and management

The N addition experiment was carried out since 2014. All experimental plots (10 m × 10 m) were set and separated by walkways. Totally random block design was adopted. Nitrogen fertilizer was mixed with roasted fine sand, and then applied evenly on the test field. The N compounds were applied annually when grasslands turned green. In this study, three nitrogen addition types were selected based on atmospheric nitrogen deposition components: NH_4_HCO_3_ (AC), NH_4_NO_3_ (AN) and (NH_4_)_2_SO_4_ (AS). Each N fertilizer was applied at six dosages: 0, 2, 5, 10, 20, and 50 g N m^−2^ yr^−1^ (nitrogen addition dosages: N0, N2, N5, N10, N20, N50). Eighteen treatments were performed, and each treatment was repeated eight times, giving 144 plots.

### 2.3 Sampling and the determination of nitrogen and phosphorus content in leaves

Plant sampling was conducted during the green and senescent seasons (August and November) in 2020. An appropriate number of intact plants with similar growth conditions of each species were collected. Then the plants were placed in sealed bags with mark, and temporarily stored in an insulation bucket in iceboxes. After sampling, the plants were immediately brought to the laboratory. Then the stems and leaves were separated and dried for 48 h at 65°C. We used the Kjeldahl apparatus (K9860, Hanon, Dezhou, China) to determine the leaf total N content (LN) after extraction with sulfuric acid, and used a UV-spectrophotometer (UA-5500, METASH, Shanghai, China) to determine the leaf total P content (LP) with the wavelength set to 700 nm. All trait measurements were based on previous standardized measurements (Pérezharguindeguy et al., 2016). Mass loss during leaf senescence can also lead to a significant underestimation of nutrient resorption, which we corrected for using a mass loss factor (MLCF) (Vergutz et al., 2012).

Leaf nitrogen resorption (NRE) was defined as the percentage of N absorbed in senesce, and calculated as the ratio of the difference in green leaves (Ng) and senescent leaves (Ns) to Ng:


(1) $$\begin{array}{c} NRE\;\left(\%\right)=\frac{\text{Ng}-\text{Ns}*\text{MLCF}}{\text{Ng}} \end{array}$$

Leaf Phosphorus resorption (PRE) is related to senescing leaf P concentration (Ps) and green leaf P concentration (Pg), using the following equation:


(2) $$\begin{array}{c} PRE\;\left(\%\right)=\frac{\text{Pg}-\text{Ps}*\text{MLCF}}{\text{Pg}} \end{array}$$

However, we found no evidence of stem mass loss during senescence, and we ignored stem mass loss. Stem nitrogen resorption efficiency (SNRE) is related to senescing stem N concentration (SNs) and green stem N concentration (SNg), using the following equation:


(3) $$\text{SNRE }\left(\%\right)=\frac{\text{SNg}-\text{SNs}}{\text{SNg}}$$

Stem phosphorus resorption efficiency (SPRE) is related to senescing stem P concentration (SPs) and green stem P concentration (SPg), using the following equation:


(4) $$\text{SPRE }\left(\%\right)=\frac{\text{SPg}-\text{SPs}}{\text{SPg}}$$

Furthermore, [N]s has also been used as an indicator for the NRP as it indicates the degree of depletion of nutrients during leaf senescence.

### 2.4 Setup of litter decomposition experiment

The most dominant species *L. chinensis* was selected for litter decomposition experiments. Decomposition experiments were conducted with garbage bags placed in the microcosm. A plastic cup (6 cm in diameter, 5 cm in height) was filled with 50 g of soil. The soil moisture content was adjusted to 20% with distilled water. All of the microscopic worlds were stored in a lab with a consistent humidity and temperature. Then 1 g of litter was put into a nylon bag and placed on the top of the soil to ensure that the litter was completely contact with the soil. Set three cups for per litter sample and controlled the temperature at 22°C and the humidity at 20%. Took samples after one month, three months, and six months. After litter recovery, the surface soil was washed with water, dried for 48 h at 65°C and then weighed the mass remaining. The rate of decomposition is expressed in terms of the litter mass remaining, the higher the residue the lower the rate of decomposition.

### 2.5 Statistical methods

All variables were tested for normality of distribution and homogeneity before analysis. We used three-way analysis of variance (ANOVA) to test the effects of N dosage, N compounds type, species, and their interactions on plant nutrient content and nutrient resorption. One-way ANOVA was used to test the differences among treatments within species, which was performed at *α* = 0.05. A Linear regression analysis was used to analyze the relationship between nitrogen addition and resorption.

Similarly, three-way analysis of variance was used to study the effects of nitrogen addition, nitrogen compound type, sampling period and their interaction on litter surplus. Under the condition of *α* = 0.05, one-way ANOVA was used to test the difference among different treatments. We used SPSS 23.0 (IBM Corp., Armonk, NY, USA) for data analyses and Origin 2021 (Originlab Co., Northampton, MA, USA) for figures.

## 3 Results

### 3.1 Effects of N addition on nutrient uptake

**Figure 1:**
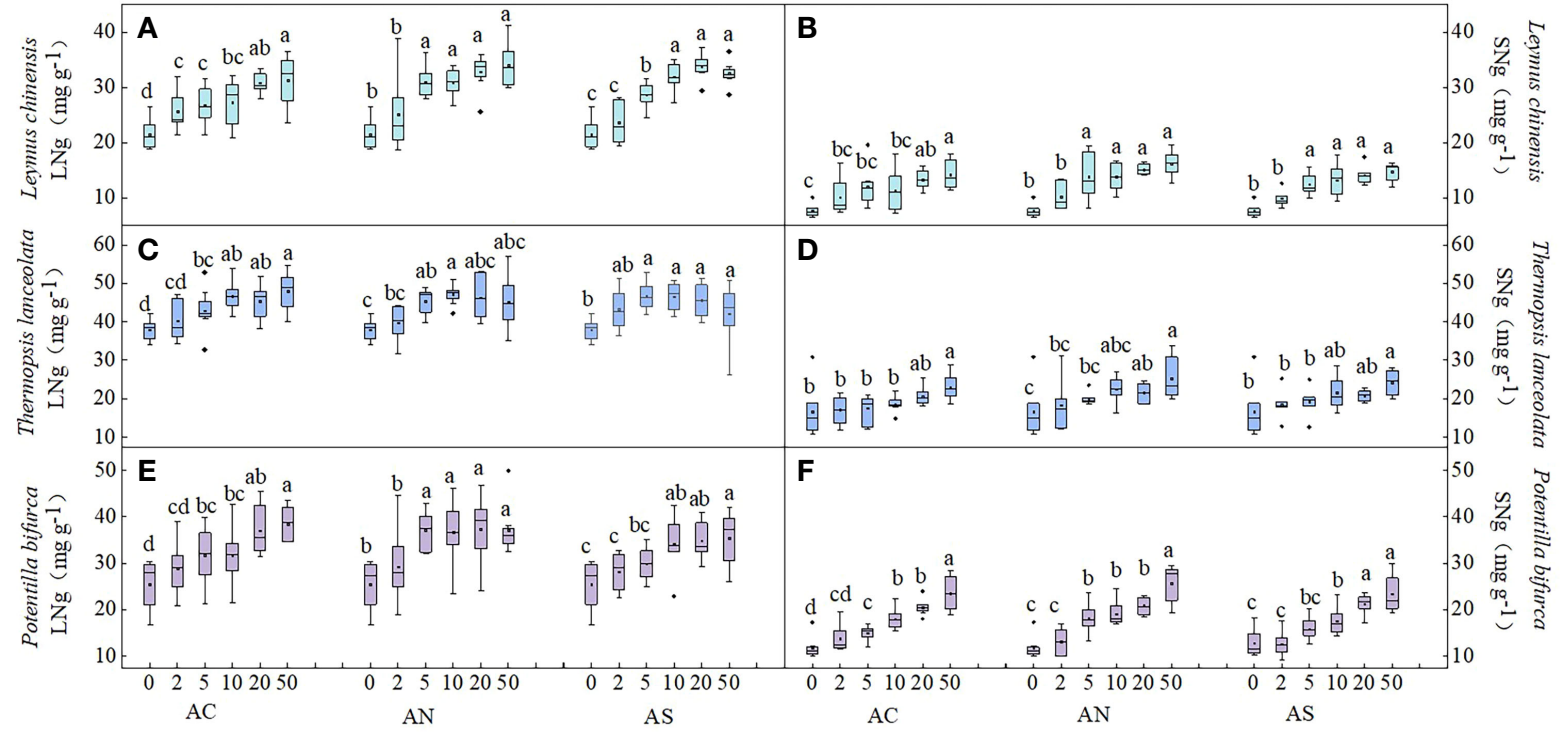
Effects of nitrogen deposition on LNg and SNg of three species. Different lowercase letters represent significant differences among treatments under the same nitrogen compound types (Duncan’s test, n = 8, P < 0.05). **(A)** represents the LNg of Leymus chinensis. **(B)** represents the SNg of Leymus chinensis. **(C)** represents the LNg of Thermopsis lanceolata. **(D)** represents the SNg of Thermopsis lanceolata. **(E)** represents the LNg of Potentilla bifurca. **(F)** represents the SNg of Potentilla bifurca.

In this study, three-factor analysis of variance was used to explore the effects of nitrogen dosage (D), nitrogen addition type (T), species (S) and their interactions on plant nutrient content and nutrient resorption efficiency. Plant nutrient content was used to represent nutrient uptake. The results showed that species and nitrogen dosage had significant effects on LNg (*P*< 0.05), the nitrogen addition type and its interaction had no significant effect on LNg (**Table 1**). Under the three nitrogen addition types, the LNg of the three species increased with the increase of nitrogen addition dosage. The LNg of the three plants decreased or remained unchanged when nitrogen addition dosages were 20 and 50 g N m^−2^ yr^−1^ (**Figures 1A, C, E** **)**. In terms of species diversity, the LNg of the leguminous plant *T. lanceolata* was the highest (**Figure 1C**).

**Table 1:** Results of three-way analysis of variance (ANOVA) for the effects of N addition dosage (D), N compounds type (T), species (S) and their interactions on plant nutrient contents and the statuses of nutrient resorption efficiency.

Parameters	S	D	T	S*D	S*T	D*T	S*D*T
**LNg**	574.08^***^	31.453^***^	2.587	0.584	0.106	1.229	0.87
**SNg**	151.85^***^	11.913^***^	4.753^*^	1.481^***^	0.511	0.470	0.206
**LNs**	1.406	7.490^***^	2.635	6.058	0.496	1.070	0.781
**SNs**	1028.2^***^	5.388^***^	2.856	1.568	0.128	0.707	1.398
**LPg**	201.72^***^	2.318^*^	2.440	3.120^*^	0.682	2.025	1.933
**SPg**	29.200^***^	3.056^*^	3.631^*^	0.850	0.559	0.287	0.265
**LPs**	30.072^***^	0.398	0.054	1.113	0.184	0.723	0.348
**SPs**	37.945^***^	0.684	0.131	0.383	1.010	0.299	0.39
**LNRE**	94.683^***^	1.071	0.538	2.468^*^	0.169	0.546	0.414
**SNRE**	42.233^***^	3.464^**^	0.679	1.288	0.145	0.354	1.03
**LPRE**	45.788^***^	1.811	0.926	0.683	0.140	0.528	0.426
**SPRE**	0.052	0.877	2.822	0.859	0.058	0.557	0.301

Asterisks denote significant levels: ns, P > 0.05; *, P< 0.05; **, P< 0.01; and ***, P< 0.001, respectively. n = 8. LNg indicates green leaf nitrogen, LNs indicates senescent leaf nitrogen, LPg indicates green leaf phosphorus, LPs indicates senescent leaf phosphorus, SNg indicates green stem nitrogen, SNs indicates senescent stem nitrogen, SPg indicates green stem phosphorus and SPs indicates senescent stem phosphorus. LNRE indicates leaf nitrogen resorption efficiency, SNRE indicates stem nitrogen resorption efficiency, LPRE indicates leaf phosphorus resorption efficiency and SPRE indicates stem phosphorus resorption efficiency.

Bold characters indicate significant differences.

Compared with leaves, the response of stem nitrogen content to different nitrogen addition types and nitrogen addition amounts was investigated. The results showed that species type, nitrogen dosage and nitrogen type had significant effects on SNg (*P*< 0.05) (**Table 1**). The SNg of all three species increased with the increase of N addition dosage (**Figures 1B, D, F** **)**. The SNg was the lowest when NH_4_HCO_3_ was applied (**Table 2**). In addition, the SNg were much lower than LNg in all three species (**Figure 1**).

**Table 2:** Results of a repeated-measures ANOVA for the effects of N compounds types on SNg and SPg.

		SNg			SPg		
species	dosage	AC	AN	AS	AC	AN	AS
*L. chinensis*	0	7.77 ± 0.53	7.77 ± 0.53	7.77 ± 0.53	1.11 ± 0.09	1.11 ± 0.09	1.11 ± 0.09
*L. chinensis*	2	10.15 ± 1.22	10.22 ± 0.84	9.21 ± 0.89	1.14 ± 0.18	1.36 ± 0.20	0.94 ± 0.06
*L. chinensis*	5	12.00 ± 1.26	13.86 ± 1.55	12.52 ± 0.72	1.34 ± 0.21	1.18 ± 0.23	0.98 ± 0.06
*L. chinensis*	10	11.42 ± 1.50	13.87 ± 0.89	13.21 ± 1.09	1.23 ± 0.22	0.99 ± 0.10	1.13 ± 0.13
*L. chinensis*	20	13.39 ± 0.61	15.33 ± 1.59	14.13 ± 0.64	**1.16 ± 0.11^a^**	**0.95 ± 0.10^ab^**	**0.76 ± 0.07^b^**
*L. chinensis*	50	14.24 ± 1.10	16.22 ± 0.80	14.72 ± 0.59	1.09 ± 0.12	0.87 ± 0.13	0.81 ± 0.08
*T. lanceolata*	0	16.60 ± 2.36	16.60 ± 2.36	16.60 ± 2.36	1.72 ± 0.12	1.72 ± 0.12	1.72 ± 0.12
*T. lanceolata*	2	17.18 ± 1.32	18.51 ± 2.42	18.44 ± 1.37	1.96 ± 0.20	1.62 ± 0.12	1.64 ± 0.12
*T. lanceolata*	5	17.98 ± 1.27	19.96 ± 0.55	19.26 ± 1.63	1.71 ± 0.17	1.64 ± 0.86	1.88 ± 0.15
*T. lanceolata*	10	**18.61 ± 0.71^b^**	**22.52 ± 1.15^a^**	**21.51 ± 1.51^ab^**	1.51 ± 0.66	1.47 ± 0.10	1.30 ± 0.05
*T. lanceolata*	20	20.60 ± 0.87	21.55 ± 0.91	20.87 ± 0.55	**1.49 ± 0.12^a^**	**1.29 ± 0.07^ab^**	**1.16 ± 0.10^b^**
*T. lanceolata*	50	23.07 ± 1.17	25.29 ± 2.05	24.21 ± 1.19	**1.50 ± 0.12^a^**	**1.12 ± 0.09^b^**	**1.13 ± 0.10^b^**
*P. bifurca*	0	10.46 ± 0.30	10.46 ± 0.30	10.46 ± 0.30	1.74 ± 0.04	1.74 ± 0.04	1.74 ± 0.04
*P. bifurca*	2	10.78 ± 1.46	10.73 ± 1.05	9.02 ± 1.15	1.44 ± 0.12	1.26 ± 0.18	1.51 ± 0.12
*P. bifurca*	5	11.36 ± 1.02	14.23 ± 1.64	11.62 ± 0.93	1.38 ± 0.09	1.49 ± 0.21	1.92 ± 0.42
*P. bifurca*	10	12.50 ± 1.72	14.48 ± 1.53	10.45 ± 0.62	1.33 ± 0.20	1.44 ± 0.19	1.01 ± 0.15
*P. bifurca*	20	14.93 ± 1.72	13.96 ± 1.66	13.52 ± 1.54	1.44 ± 0.20	1.40 ± 0.21	1.12 ± 0.14
*P. bifurca*	50	16.27 ± 2.42	15.82 ± 1.49	13.27 ± 1.60	1.72 ± 0.24	1.35 ± 0.15	1.14 ± 0.23

Letters indicate the difference in the nitrogen and phosphorus content of stem with different nitrogen type under various nitrogen addition dosage.

Bold characters indicate significant differences.

**Figure 2:**
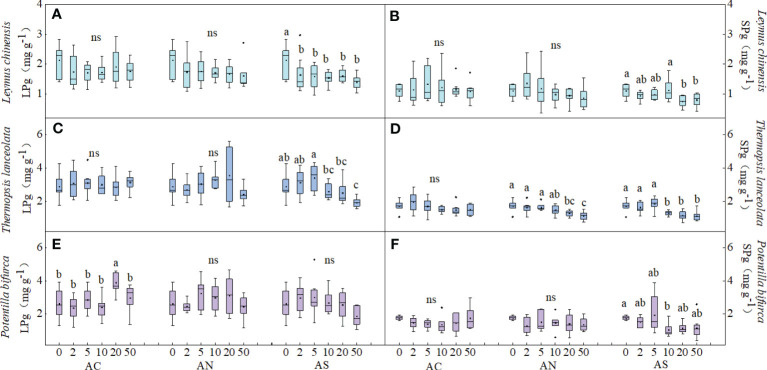
Effects of nitrogen deposition on LPg and LPs of three species. Different lowercase letters represent significant differences among treatments under the same nitrogen compound types (Duncan‘s test, n = 8, P < 0.05). **(A)** represents the LPg of Leymus chinensis. **(B)** represents the SPg of Leymus chinensis. **(C)** represents the LPg of Thermopsis lanceolata. **(D)** represents the SPg of Thermopsis lanceolata. **(E)** represents the LNg of Potentilla bifurca. **(F)** represents the SNg of Potentilla bifurca.

Species and N addition dosage had a significant effect on LPg. Species, N addition dosage and type had a significant effect on SPg (*P*< 0.05) (**Table 1**). In all species, both LPg and SPg tended to decrease or did not show a significant trend with the increase of nitrogen addition dosage. (**Figure 2**). The SPg was the highest when NH_4_HCO_3_ was applied (**Table 2**).

### 3.2 Effects of N addition on nutrient resorption

In this study, nutrient resorption efficiency was calculated by combining the nutrient contents of stems and leaves at maturity and aging stages to explore the nutrient resorption strategies adopted by different plant organs in response to the dosage and type of nitrogen deposition. The results showed that nitrogen addition dosage had a significant impact on SNRE. It had no significant effect on LNRE. Nitrogen compound type had no significant effect on LNRE and SNRE (*P*< 0.05) (**Table 1**).

**Figure 3:**
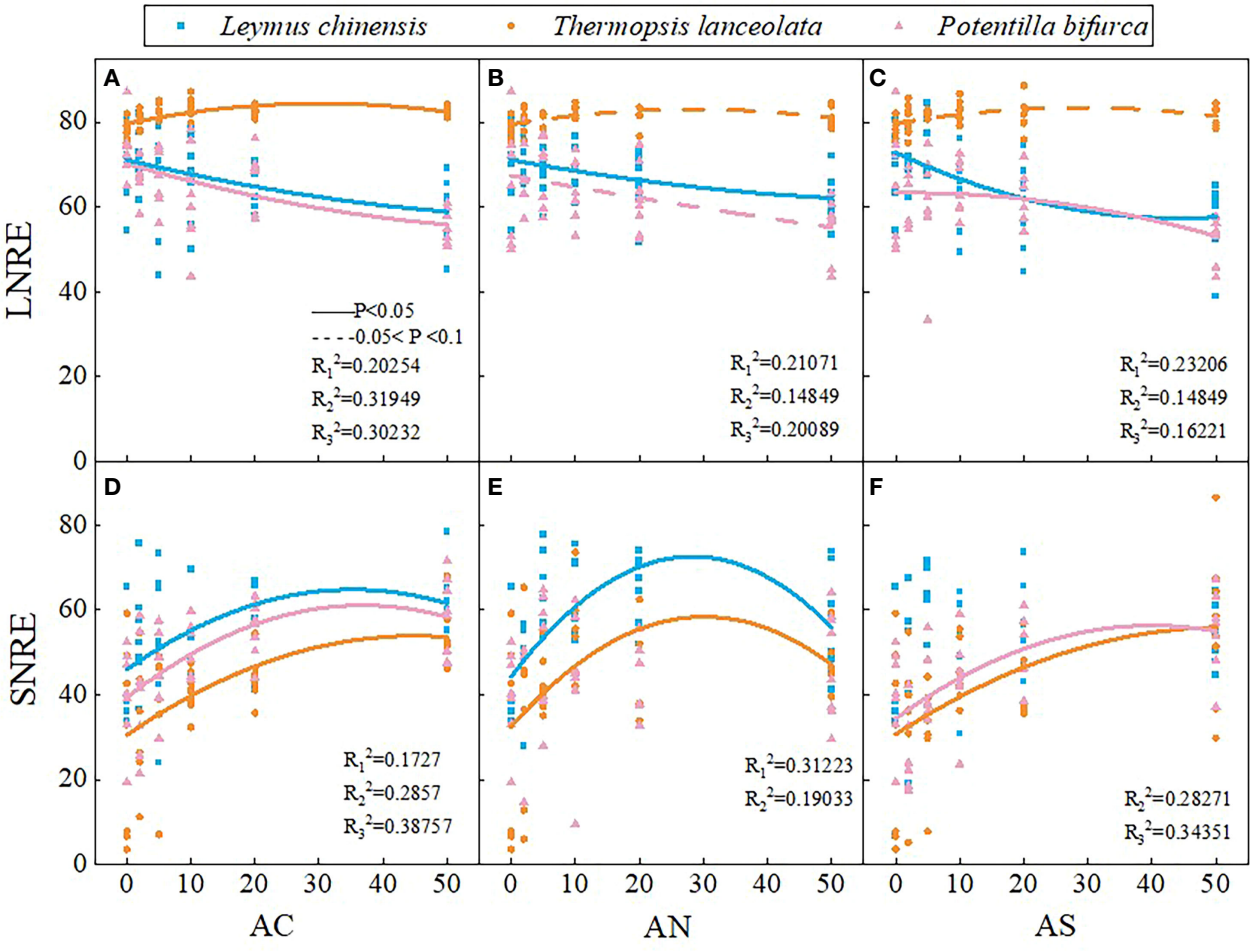
Effects of nitrogen deposition on SNRE and LNRE of three species. The solid line represents P < 0.05, and the dashed line represents 0.05<P<0.1. **(A)** represents the LNRE of Leymus chinensis. **(B)** represents the LNRE of Thermopsis lanceolata. **(C)** represents the LNRE of Potentilla bifurca. **(D)** represents the SNRE of Leymus chinensis. **(E)** represents the SNRE of Thermopsis lanceolata. **(F)** represents the SNRE of Potentilla bifurca.

With the increase of nitrogen addition dosage, the LNRE of *L. chinensis* and *P. bifurca* decreased, while the LNRE of *T. lanceolata* increased first and then decreased (**Figures 3A–C** **)**. The highest LNRE of *T. lanceolata* appeared when the supplemental dosage of nitrogen was about 30 g N m^−2^ yr^−1^ (**Figures 3A–C** **)**. In addition, the LNRE of *T. lanceolata* was higher than that of *L. chinensis* and *P. bifurca* (**Figures 3A–C** **)**. The SNRE of the three plants increased first and then decreased with the increase of nitrogen addition dosage (**Figures 3D–F** **)**. Moreover, the SNRE of *T. lanceolata* was lower than that of *L. chinensis* and *P. bifurca*, and the SNRE of *L. chinensis* was the highest (**Figures 3D–F** **)**. In addition, the LNRE was higher than that the SNRE (**Figure 3**).

**Figure 4:**
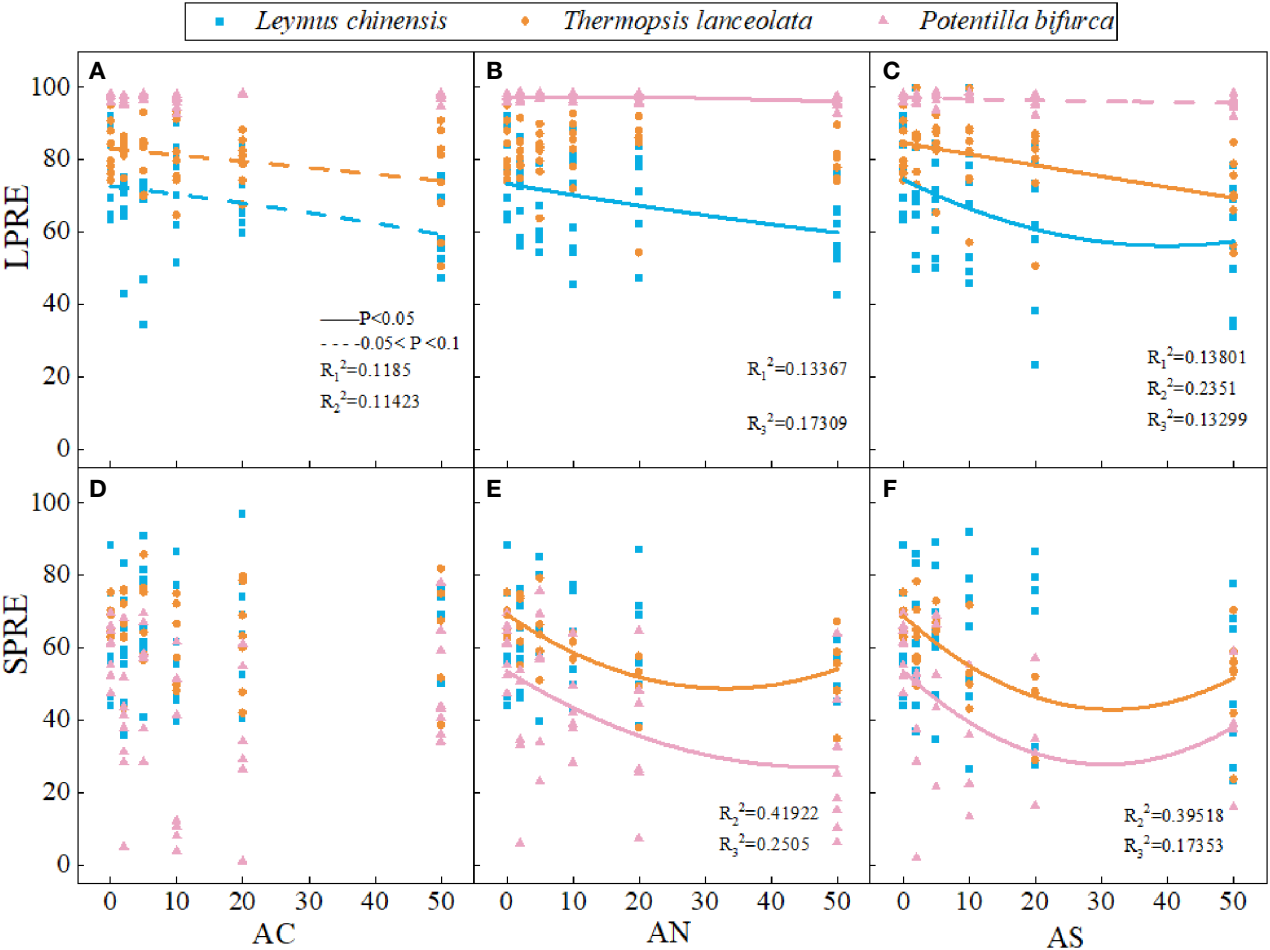
Effects of nitrogen deposition on SPRE and LPRE of three species. The solid line represents P < 0.05, and the dashed line represents 0.05<P<0.1. **(A)** represents the LPRE of Leymus chinensis. **(B)** represents the LPRE of Thermopsis lanceolata. **(C)** represents the LPRE of Potentilla bifurca. **(D)** represents the SPRE of Leymus chinensis. **(E)** represents the SPRE of Thermopsis lanceolata. **(F)** represents the SPRE of Potentilla bifurca.

Phosphorus nutrient resorption in plants is a way to make full use of phosphorus element. The results showed that nitrogen addition dosage and type had no significant effect on LPRE and SPRE (**Table 1**). LPRE decreased with the increase of nitrogen addition dosage (**Figures 4A–C** **)**. The SPRE of *T. lanceolata* and *P. bifurca* decreasing first and then increasing with the increase of nitrogen addition dosage (**Figures 4D–F** **)**. There were no significant differences in PRE between leaves and stems (**Figure 4**).

### 3.3 Effects of N addition on litter decomposition

**Figure 5:**
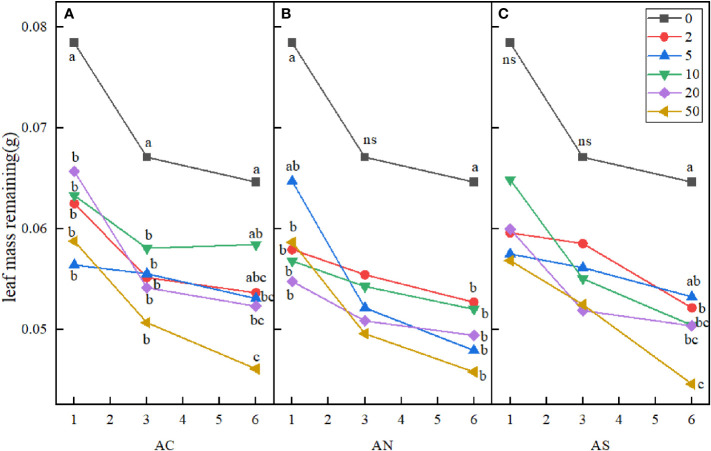
Effects of nitrogen deposition on leaf mass remaining. Different lowercase letters represent significant differences among treatments under the same date (Duncan’s test, n = 3, P < 0.05). **(A)** represents the leaf mass remaining when added NH4HCO3. **(B)** represents the leaf mass remaining when added NH4NO3. **(C)** represents the leaf mass remaining when added (NH4)2SO4.

In this study, three-factor analysis of variance was used to explore the effects of nitrogen addition dosage (D), nitrogen compound type (T), sampling period (P) and their interactions on stem and leaf litter remaining. The results showed that sampling period and nitrogen addition dosage had significant effects on leaf litter remaining, but the type of nitrogen addition had no significant effect on it (**Table 3**). Compared with the control, nitrogen addition dosage significantly increased the decomposition rate of leaf litter. At the sixth month of sampling, litter decomposition was fastest when the nitrogen addition dosage was 50 g N m^−2^ yr^−1^ (**Figure 5**).

**Table 3:** Results of a repeated-measures ANOVA for the effects of N addition dosage (D), N compounds type (T), period(P) and their interactions on leaf and stem mass remaining.

Parameters	P	T	D	P * T	P * D	T * D	P*T*D
Stem mass remaining	126.849***	5.047**	13.824***	1.498	1.073	0.699	0.716
Leaf mass remaining	55.109***	2.772	45.397***	0.235	0.989	0.676	0.809

Asterisks denote significant levels: ns, P > 0.05; **P< 0.01; and ***P< 0.001, respectively. n = 8.

Bold characters indicate significant differences.

**Figure 6:**
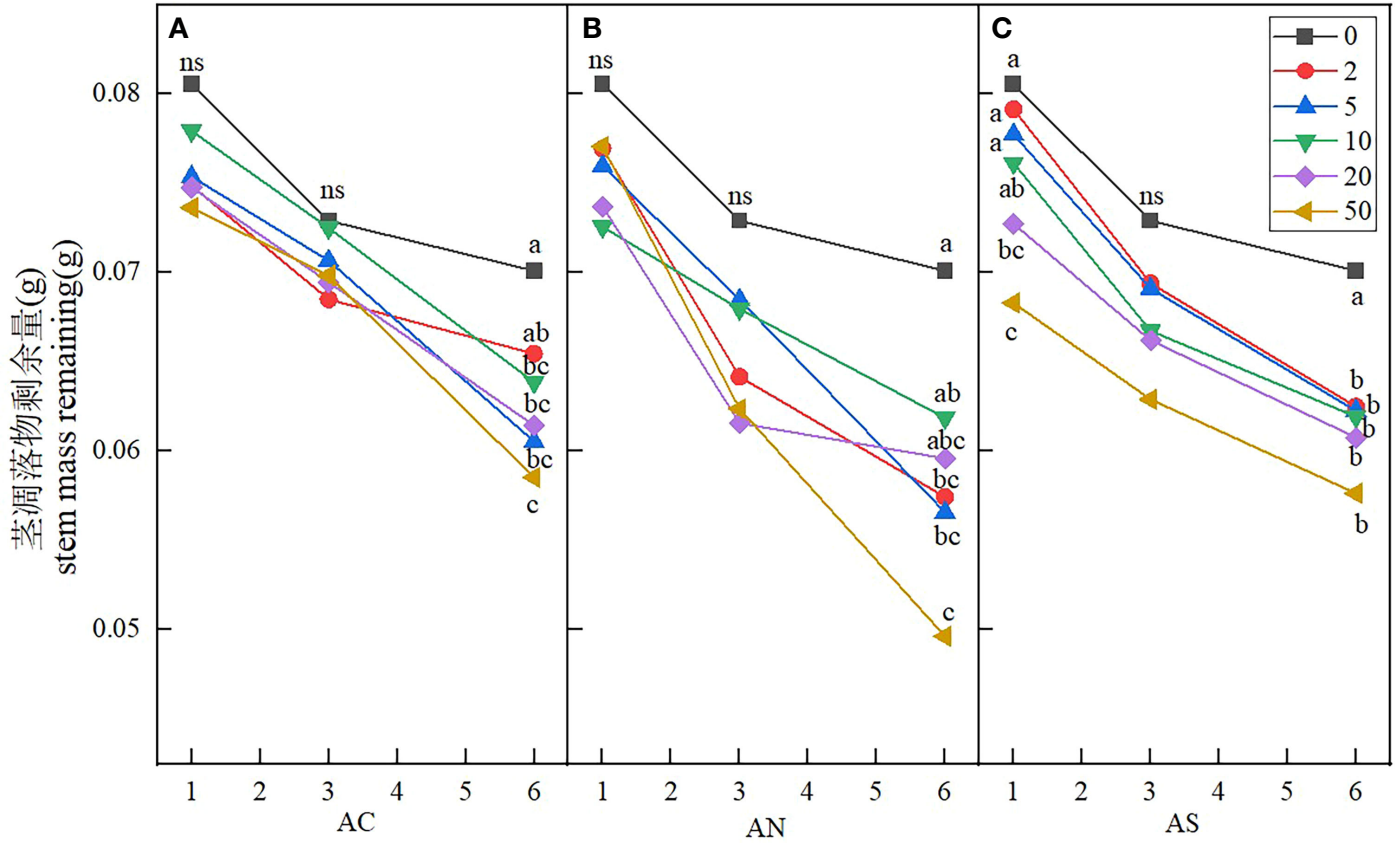
Effects of nitrogen deposition on stem mass remaining. Different lowercase letters represent significant differences among treatments under the same date (Duncan’s test, n = 3, P < 0.05). **(A)** represents the stem mass remaining when added NH4HCO3. **(B)** represents the stem mass remaining when added NH4NO3. **(C)** represents the stem mass remaining when added (NH4)2SO4.

Stem litters and leaf litters had different structure and element composition, and the response of stem litters to nitrogen addition was also different from that of leaf litters. The results showed that sampling period, nitrogen addition dosage and nitrogen compound type had significant effects on the stem litter remaining (**Table 3**). Nitrogen addition dosage significantly increased the decomposition rate of stem litter compared with the control. At the sixth month of sampling, litter decomposition was fastest when the nitrogen addition dosage was 50 g N m^−2^ yr^−1^ (**Figure 6**). The stem litter decomposition was lowest when NH_4_HCO_3_ was applied, while the litter decomposition was fast when NH_4_NO_3_ was applied (**Table 4**).

**Table 4:** Results of a repeated-measures ANOVA for the effects of N compounds types on stem mass remaining.

D	AC(1 month)	AN(1 month)	AS(1 month)	AC(3 months)	AN(3 months)	AS(3 months)	AC(6 months)	AN(6 months)	AS(6 months)
0	0.081 ± 0.0005	0.081 ± 0.0005	0.081 ± 0.0005	0.073 ± 0.002	0.073 ± 0.002	0.073 ± 0.002	0.070 ± 0.0001	0.070 ± 0.0001	0.070 ± 0.0001
2	0.075 ± 0.002	0.077 ± 0.002	0.079 ± 0.001	0.069 ± 0.001	0.064 ± 0.005	0.069 ± 0.002	0.065 ± 0.002	0.057 ± 0.005	0.063 ± 0.0004
5	0.075 ± 0.003	0.075 ± 0.003	0.078 ± 0.002	0.071 ± 0.002	0.068 ± 0.005	0.069 ± 0.001	0.061 ± 0.002	0.057 ± 0.005	0.062 ± 0.002
10	**0.078 ± 0.001^a^**	**0.073 ± 0.001^b^**	**0.076 ± 0.001^ab^**	0.073 ± 0.004	0.068 ± 0.005	0.067 ± 0.004	0.064 ± 0.002	0.062 ± 0.001	0.062 ± 0.003
20	0.075 ± 0.002	0.074 ± 0.001	0.073 ± 0.004	0.069 ± 0.0004^a^	0.062 ± 0.001^c^	0.066 ± 0.001^b^	0.061 ± 0.001	0.060 ± 0.003	0.061 ± 0.002
50	0.074 ± 0.005	0.077 ± 0.004	0.068 ± 0.003	0.070 ± 0.005	0.062 ± 0.001	0.063 ± 0.005	0.059 ± 0.001	0.050 ± 0.003	0.058 ± 0.005

Letters indicate the difference in litter decomposition of plants with different nitrogen type under various nitrogen addition dosage. 1 month, 3 months and 6 months represent samples taken one month, three months and six months after decomposition respectively.

Bold characters indicate significant differences.

## 4 Discussion

### 4.1 Effects of N addition on plant nutrient uptake

Nitrogen deposition regulates plant nitrogen and phosphorus stoichiometry by changing soil nutrient content and plant nutrient use efficiency (Cleveland et al., 2011; Li et al., 2016). The results of this study showed that nitrogen content in mature stems and leaves of the three species increased with the increase of nitrogen addition dosage, which was consistent with the results of previous studies (Lü et al., 2013). Theoretically, increased application of nitrogen fertilizer can significantly improve soil nitrogen availability (Ladwig et al., 2011; Fu and Shen, 2017), which extensively promotes plant nitrogen concentration. However, they decreased slightly at the N addition dosages of 20 and 50g Nm^−2^yr^−1^, which may be caused by the ammonia toxicity due to excess N fertilizer (Berg et al., 2005). The stem nitrogen content was the lowest when NH_4_HCO_3_ was applied, which probably due to that NH_4_HCO_3_ is not suitable for shallow application, as it could easy to decompose into NO_3_ and volatilize (Liu et al., 1997).

The results of this study showed that phosphorus content in green stems and leaves showed a decreasing trend or no significant difference with the addition dosage of nitrogen in all species. Studies have shown that the addition of nitrogen fertilizer significantly increased the nitrogen concentration of plants, but often reduced the phosphorus concentration in green leaves (Yuan and Chen, 2015). This phenomenon can be simply explained as that nitrogen application significantly increases the availability of soil nitrogen (Ladwig et al., 2011; Fu and Shen, 2017), which extensively promotes the nitrogen concentration of plants, and the growth of plants will lead to the increase of biomass, thus diluting the phosphorus concentration of plants (Agren, 2004). In previous studies, the response of phosphorus concentration to nitrogen addition was significantly different, and some studies showed negative (Menge and Field, 2007) or no significant effect (Chen et al., 2015), which was consistent with the results of our study. This controversy may be attributed to nutrient restriction conditions (including nitrogen restriction, nitrogen and phosphorus co-restriction, and phosphorus restriction), which may affect the response of plants to nitrogen and phosphorus uptake and nitrogen and phosphorus loss. Nutrient restriction conditions determined the plant nitrogen and phosphorus stoichiometric response to nitrogen addition and could be regulated by plant growth type. In addition, under different nutrient restriction conditions, the responses of plant nitrogen and phosphorus stoichiometry to nitrogen addition are strongly influenced by geographical factors, climatic factors and experimental conditions (You et al., 2018).

### 4.2 Effects of N addition on plant nutrient resorption

With the addition of nitrogen, the availability of N in the soil increased and plants relied more on taking nutrients from the soil. The proportion of N obtained by nutrient resorption in N-rich environments was reduced, so the LNRE of *L. chinensis* and *P. bifurca* decreased (Shi et al., 2020). However, the LNRE of *T. lanceolata* increased first and then decreased. The availability of N and P could influence the nitrogen fixation by varying the biomass of grass legumes, and the contribution of N from symbiotic N fixation to the total N content of legumes. Nitrogen inputs could affect the symbiotic nitrogen fixation of legume plants in grasslands, and nitrogen fixation capacity generally decreases with availability of both ammonium and nitrate in soil. Legumes could preferentially take up active nitrogen from the soil in N-rich environment because the high energetic cost of N-fixation (Tognetti et al., 2021; Vázquez et al., 2022). Therefore, with the addition of nitrogen, the increase of LNRE of *T. lanceolata* may be due to the decrease of the symbiotic nitrogen fixation ability caused by the addition of nitrogen. At the later stage, the dependence on resorption gradually decreased with the increase of soil N availability.

The SNRE of the three species showed a trend of increasing and then decreasing. Previous studies had shown that higher green organ nutrient concentrations generally lead to lower nutrient resorption efficiencies worldwide (Li et al., 2015). In addition, the nitrogen resorption efficiency of leaves was greater than that of stems, which may be due to lower nutrient concentrations in stems than in leaves (Mao et al., 2013). This was partly attributable to the higher nutrient content of leaves and compared with stems, and probably due to the higher percentage of hydrolysable compounds (mainly proteins) in the leaves than in the stems, and the higher amount of structural compounds in the stems is less soluble due to their supporting functions (Freschet et al., 2010). Therefore, due to the low nitrogen concentration in the early stage, the nutrient resorption efficiency of the stem increased first and then decreased as the N addition.

In previous studies, the relationship between N availability and P resorption efficiency remained uncertain, and the P resorption efficiency increased (Shi et al., 2020) or decreased (Lü et al., 2013) or remained unchanged (Kozovits et al., 2007) after N application. In our study, the response of leaves and stems to N addition was different. In leaves, PRE decreased with the increase of nitrogen application. The results showed that the addition of N accelerated the uptake and turnover of N and P in temperate grassland ecosystems, and the N and P cycles were dynamically coupled (Agren et al., 2012). The convergent (both decreasing) response of N and P resorption to N addition suggested a stoichiometric coupling of N and P resorption processes at the intraspecies level among dominant grasses in temperate grasslands and provides a mechanism for increasing N and P limitation in most terrestrial ecosystems (Elser et al., 2007). While in stems PRE showed a trend of increasing and then decreasing. Therefore, it is necessary to take into account both leaf and non-leaf organs in order to fully understand the effect of N concentration on the resorption of P in grassland.

### 4.3 Effects of N addition on litter decomposition

With the increase of nitrogen addition dosage, the initial nitrogen content of litter increases, the litter decomposition increased. Previous studies had shown that litter decomposition was mainly controlled by the concentration of N in litter, and litter with high N content was more likely to decompose (Liu et al., 2022). The decomposition of plant debris is mainly carried out by bacteria and fungi, which have relatively high N and P contents, indicating a high demand for these nutrients. Therefore, high-N and high-P litter will decompose more quickly than low-nutrient litter, since high quality litter can stimulate microbial population growth (Li et al., 2017). Some studies predicted that compounds in high-quality waste were more likely to degrade than low-quality waste, because the decomposition of low-quality waste required specialized decomposers (Ayres et al., 2009). Substrate N was positively correlated with decomposition, suggesting that nitrogen chemistry were important aspects of litter quality for decomposers at the early stages of decomposition (Hobbie, 2005).

The type of N application had no significant effect on leaf litter, but did have an effect on stem litter decomposition, especially when NH_4_NO_3_ was applied, which had a faster rate of stem litter decomposition. This may be due to the fact that compared with ${\text{NH}}_{4}^{+}$ , ${\text{NO}}_{3}^{-}$ had a higher diffusion coefficient in soil, and the addition of NH_4_NO_3_ has the highest promotion effect on soil respiration (Diao et al., 2022).

## 5 Conclusion

In conclusion, this study quantitatively assessed the response of nutrient uptake, nutrient resorption and litter decomposition to nitrogen deposition. Our results suggested that nitrogen deposition can greatly affect ecosystem nutrient cycle of nitrogen and phosphorus. Nitrogen deposition dosage increased nitrogen uptake but decreased phosphorus uptake of stems and leaves. Besides, nitrogen addition type had a significant effect on nitrogen and phosphorus content of stems. Nitrogen addition dosage had a significant effect on plant nutrient resorption, while nitrogen addition type had no significant effect on it. Otherwise, nitrogen deposition dosage accelerated litter decomposition. Nitrogen application type had significant effect on stem litter decomposition. These results clearly showed that different plant organs had different responses to nitrogen deposition, and the nitrogen deposition types also affect plant nutrient cycling.

## Data availability statement

The original contributions presented in the study are included in the article/**Supplementary material**. Further inquiries can be directed to the corresponding author.

## Author contributions

YangZ, HW, PZ, and RW proposed the study and designed the experiment. YZ conducted field and laboratory measurements, and analyzed the data. HW, PZ, and RW secured fundings. QZ, WY, YanZ, NW, PF, CY, LY, QG, HW, PZ, and RW helped with laboratory measurements and data analysis. NW and WY helped with data analysis. YangZ wrote the manuscript that was intensively edited by all authors.
