# Supplementary material for: Response mechanisms of 3 typical plants nitrogen and phosphorus nutrient cycling to nitrogen deposition in temperate meadow grasslands
Source: Front Plant Sci. 2023 Jul 7;14:1140080. doi: 10.3389/fpls.2023.1140080 (PMC10361690; doi:10.3389/fpls.2023.1140080)
Supplement: Supplementary file 1 [file DataSheet_1.zip › Supplementary Tables and Figures.pdf]

Supplementary materials

Table S1 Spearman's correlation coefficients of leaf initial chemical properties, nutrient resorption and mass remaining.

	initial N	Initial P	LNRE	LPRE	mass (1 mouth)	mass (3 mouths)	mass (6 mouths)
initial N	1	**0.728****	**-0.863****	**-0.750****	**-0.318***	**-0.305***	**-0.399****
Initial P		1	**-0.739****	**-0.848****	-0.281	-0.085	-0.079
LNRE			1	**0.751****	0.193	0.160	0.243
LPRE				1	0.273	0.151	0.254
mass (1 mouth)					1	**0.477****	**0.296***
mass (3 mouths)						1	**0.470****
mass (6 mouths)							1

Note: Values in bold mean significant correlation: *P < 0.05, ** P < 0.01.

Table S2 Spearman's correlation coefficients of stem initial chemical properties, nutrient resorption and mass remaining.

	initial N	Initial P	LNRE	LPRE	mass (1 mouth)	mass (3 mouths)	mass (6 mouths)
initial N	1	**0.288****	**-0.863****	**-0.458****	-0.204	-0.099	-0.234
Initial P		1	-0.288	**-0.559****	0.237	0.115	-0.017
SNRE			1	**0.337***	**-0.448****	**-0.329***	-0.080
SPRE				1	-0.118	-0.207	0.103
mass (1 mouth)					1	0.253	0.112
mass (3 mouths)						1	0.256
mass (6 mouths)							1

Note: Values in bold mean significant correlation: *P<0.05, **P<0.01

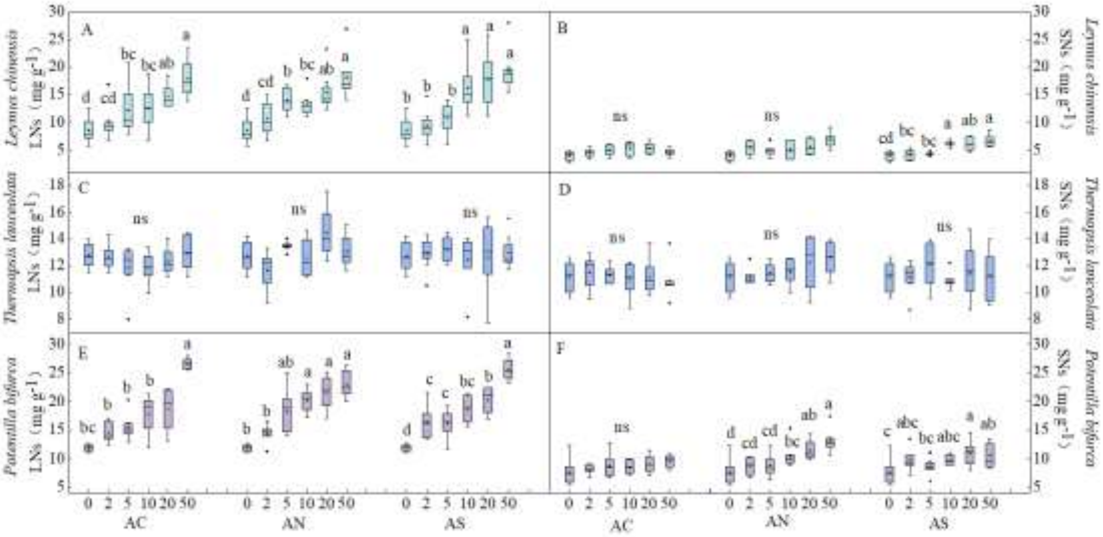

Fig. S1 Effects of nitrogen deposition on LNs and SNs of three species. Different lowercase letters represent significant differences among treatments under the same nitrogen compound types (Duncan's test, n = 8, P < 0.05).

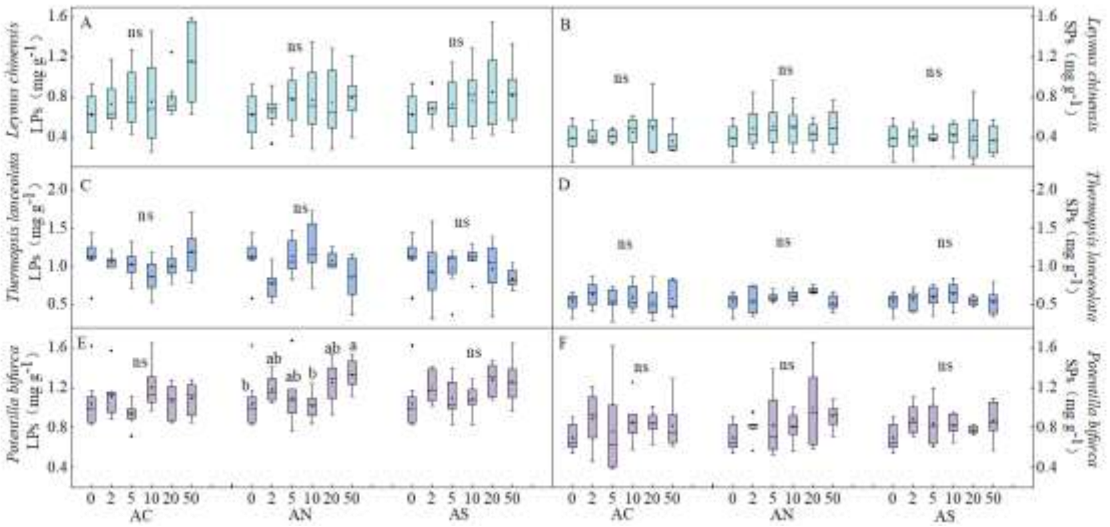

Fig. S2 Effects of nitrogen deposition on SPg and SPs of three species. Different lowercase letters represent significant differences among treatments under the same nitrogen compound types (Duncan's test, $n = 8$, $P < 0.05$).
